# Motivational Interventions for Reducing Excessive Alcohol Consumption Among University Students: A Systematic Review and Meta-Analysis

**DOI:** 10.3390/healthcare13192405

**Published:** 2025-09-24

**Authors:** Víctor Serrano-Fernández, Esperanza Barroso-Corroto, Cristina Rivera-Picón, Brigida Molina-Gallego, Ana Quesado, Juan Manuel Carmona-Torres, Pablo Jesús López-Soto, Alba Sánchez-Gil, Juan Luis Sánchez-González, Pedro Manuel Rodríguez-Muñoz

**Affiliations:** 1 Facultad de Fisioterapia y Enfermería, Universidad de Castilla-La Mancha, 45071 Toledo, Spain; victor.serrano3@alu.uclm.es (V.S.-F.); esperanza.barroso@uclm.es (E.B.-C.); brigida.molina@uclm.es (B.M.-G.); juanmanuel.carmona@uclm.es (J.M.C.-T.); pedrom.rodriguez@uclm.es (P.M.R.-M.); 2 Facultad de Ciencias de la Salud, Universidad de Castilla-La Mancha, 45600 Talavera de la Reina, Spain; 3 Escola Superior de Saúde, Universidade de Aveiro, 3810-193 Aveiro, Portugal; anaquesado@ua.pt; 4 Facultad de Medicina y Enfermería, Universidad de Córdoba, 14071 Córdoba, Spain; n82losop@uco.es; 5 National Centre of Reference for People with Alzheimer’s Disease and Other Dementias, 37008 Salamanca, Spain; asanchezzg02@gmail.com; 6 Departamento de Enfermería y Fisioterapia, Universidad de Salamanca, 37007 Salamanca, Spain; juanluissanchez@usal.es

**Keywords:** motivational interviewing, university students, alcohol consumption, prevention

## Abstract

**Background/Objectives:** University students frequently engage in risky alcohol consumption, making them a priority population for targeted interventions. Motivational interventions (MIs) have been widely implemented to address this issue, but evidence of their effectiveness remains heterogeneous. This study aimed to evaluate the efficacy of MIs in reducing alcohol consumption and related harm among university students through a systematic review and meta-analysis. **Methods:** A systematic search was conducted in PubMed, Scopus, and BVS Library, including randomized controlled trials (RCTs) published up to April 2025. The PRISMA and RoB-2 tools guided reporting and risk of bias assessment. Random-effects models were applied to pool effect sizes for changes in alcohol consumption patterns and related consequences. **Results:** Fifteen RCTs were included. MIs significantly reduced daily alcohol intake (−0.55 drinks/day; 95% CI: −0.78 to −0.32), with additional reductions in weekly consumption and binge drinking episodes, though these were not statistically significant. Positive effects were also observed in reducing alcohol-related consequences and blood alcohol concentration levels. Short, single-session formats (45–90 min) showed consistent efficacy across studies, with effects sustained at 2–3 months and, in some cases, up to one year post-intervention. **Conclusions:** MIs are effective, brief, and adaptable strategies for reducing harmful alcohol use and associated negative outcomes among university students. Their simplicity, feasibility, and sustained effects make them valuable tools for university health programs. Future research should focus on optimizing intervention components and evaluating their effectiveness in diverse cultural and socioeconomic contexts.

## 1. Introduction

The World Health Organization (WHO), whose main objective is to promote health, safeguard global security, and support vulnerable populations, provides alarming data on alcohol consumption. According to its reports, alcohol use contributes to death and disability from an early age and accounts for 13.5% of all deaths among individuals aged 20 to 39 years [1]. Alcohol consumption is widespread and culturally accepted in most European countries, currently representing a major public health concern [2].

A recent meta-analysis estimates that 155 million university students consume alcohol, with the highest prevalence found in Europe (43.8%), the Americas (38.2%), and the Western Pacific (37.9%). In the United States, 30.7% of university students reported alcohol consumption, compared to 27.4% among their non-university peers. In African and Asian countries, prevalence varies significantly: Egypt, 2.7%; Tunisia, 52.5%; and Ethiopia, 35.5% [3]. In Spain, alcohol consumption has traditionally been associated with adulthood and characterized by regular use, its integration into dietary habits, and its role in social events. However, recent years have seen notable changes have occurred in both the quantity consumed and the patterns and meanings associated with alcohol use [4]. Today, alcohol consumption is considered one of the leading risk factors for disability and premature death [5].

Among university students, alcohol consumption represents a significant public health concern. Several studies have shown that over 80% of university students consume alcohol, and approximately 40% have engaged in episodes of excessive drinking in the weeks prior to assessment [6]. This drinking pattern, commonly referred to as binge drinking, has been linked to serious negative outcomes, including poor academic performance, risky sexual behaviors, legal issues, interpersonal violence, and even alcohol-related deaths [7,8].

The latest report from the European School Survey Project on Alcohol and Other Drugs (ESPAD) indicates that 34% of young people reported engaging in binge drinking [9]. In Spain, according to the most recent Survey on Alcohol and Other Drugs (EDADES), 57.9% of young people reported alcohol consumption in the past 30 days [10].

Given these findings, alcohol use among university students has become an increasingly pressing issue in public health, due to both its high prevalence and the adverse consequences it entails at the individual, academic, and social levels. Over recent decades, various studies have shown that this behavior—traditionally accepted within the European cultural context—has undergone significant transformations in terms of consumption patterns, frequency, and intensity, particularly among young people in higher education [11]. These changes include an increase in the consumption of high-alcohol-content beverages and the normalization of practices such as excessive drinking in leisure settings, underscoring the urgent need for effective intervention and prevention strategies.

In this context, motivational interventions (MIs) have proven to be an effective and cost-efficient strategy for reducing harmful alcohol consumption among young people. These interventions aim to resolve ambivalence, enhance intrinsic motivation, and empower behavioral change [12]. Modalities such as motivational interviewing and brief personalized programs have demonstrated effectiveness when tailored to the typical drinking patterns and underlying motives characteristic of this life stage [13]. Unlike traditional informational approaches, MI incorporates psychological, social, and cultural factors that influence behavior, offering responses that are better aligned with the needs of the university environment [14].

Brief MIs based on motivational interviewing have been shown to be effective in reducing alcohol consumption among young populations, particularly when adapted to the specific characteristics and needs of the university context [15]. Motivational interviewing is a client-centered counseling style designed to enhance intrinsic motivation for change by exploring ambivalence and reinforcing self-efficacy [16]. Core strategies include open-ended questioning, reflective listening, and collaborative goal setting. Among university students, MI are typically brief, delivered individually or in groups, and often incorporate personalized normative feedback [15,17,18]. MI is a therapeutic approach that fosters change through an empathetic, non-confrontational, and collaborative relationship [16]. In its group-based application—whether as group motivational enhancement therapy or as a brief intervention in educational setting—significant reductions have been observed in alcohol consumption, the number of intoxication episodes, and alcohol-related problems [17,18].

One of the most promising approaches to understanding and addressing this issue is the study of the underlying motives for alcohol consumption. From a motivational perspective, research has shown that university students consume alcohol for various reasons, ranging from the pursuit of social and emotional gratification to coping with negative emotional states such as anxiety or depression [19]. The five-factor model proposed in the Modified Drinking Motives Questionnaire-Revised (M DMQ-R) distinguishes between motives based on positive reinforcement (social and enhancement) and those based on negative reinforcement (conformity and coping). This distinction is essential for designing interventions tailored to the needs and characteristics of the target group.

Moreover, cross-cultural studies have demonstrated that, despite contextual differences between countries, drinking motives exhibit a consistent structure. This suggests that prevention strategies focused on these factors could be generalized across different university settings [19]. However, as highlighted in previous research, mere awareness of the risks associated with alcohol consumption does not necessarily lead to a meaningful reduction in its incidence. This reinforces the importance of targeting the specific motivations that sustain this behavior [11]. Therefore, it is necessary to evaluate the effectiveness of motivational interviewing-based interventions in reducing problematic alcohol use among university students.

This review builds upon previous evidence syntheses by: (i) restricting eligibility to randomized controlled trials (RCTs) to maximize internal validity; (ii) incorporating studies published after 2014, including contemporary delivery formats such as brief and digitally supported MI; and (iii) conducting outcome-specific meta-analyses—of daily/weekly intake, binge episodes, alcohol-related consequences measured by RAPI/YAACQ, and blood alcohol concentration—using standardized methods. MIs are particularly necessary in university settings, where alcohol use is often driven by complex emotional and social factors that traditional informational approaches fail to address. MIs offer a personalized and empathetic strategy that targets these underlying motives, making it especially suitable for this population [11,16,17,18,19]. By covering the literature up to 30 April 2025, this review provides the most current, trial-based estimate of MI effectiveness among university students and identifies remaining research gaps to inform the development of next-generation interventions.

## 2. Materials and Methods

### 2.1. Study Design and Data Sources

This study corresponds to a systematic review with meta-analysis conducted on RCTs. The review followed the guidelines established by the *Preferred Reporting Items for Systematic Reviews and Meta-Analyses* (PRISMA) checklist [20].

The databases used for study retrieval were PubMed, Scopus, and the Virtual Health Library (BVS). Search covered the period from database inception to 30 April 2025.

### 2.2. Search Strategy

The searches were conducted from inception to 30 April 2025 to answer the following research question framed using the PICO format (Population, Intervention, Control, Outcome): *What is the effectiveness of motivational interventions in reducing alcohol consumption among university students compared to no intervention or other types of interventions?*

The detailed search strategy (including MeSH terms, Boolean operators, and filters applied across the different databases) used to address this question is presented in Table 1.

### 2.3. Inclusion and Exclusion Criteria

The following inclusion criteria were applied to select studies for this review:

- RCTs.
- Samples consisting of university students.
- Study objective: addressing alcohol consumption.
- Language: Spanish or English.

The exclusion criteria were as follows:

- Non-university populations.
- Studies evaluating the consumption of other substances.
- Grey literature, narrative reviews, systematic reviews, and meta-analyses.

We restricted our inclusion to RCTs to maximize internal validity and minimize selection bias. Pre-experimental and non-randomized designs were excluded because of their higher risk of bias and limited methodological rigor.

### 2.4. Study Selection Process

**Figure 1:**
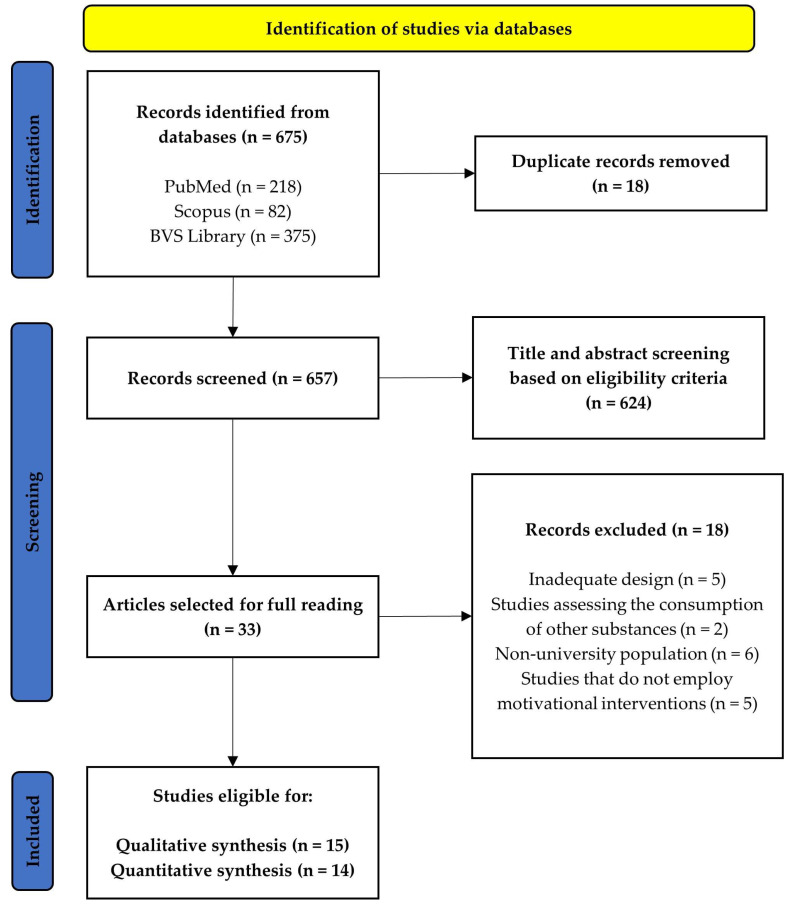
PRISMA flow diagram.

Two reviewers (VSF and PMRM) independently screened titles/abstracts and full texts against the predefined inclusion and exclusion criteria. Duplicate records were removed using Mendeley Reference Manager. At the title/abstract stage, potentially eligible studies were retained for full-text review. Reasons for exclusion at full text (e.g., inadequate design, wrong population, non-motivational intervention, or insufficient outcome data) were documented and are summarized in the PRISMA flow diagram (Figure 1) Discrepancies between reviewers were resolved by discussion and consensus, with adjudication by a third reviewer (CRP) when necessary.

### 2.5. Risk of Bias Assessment

Risk of bias was assessed using the *Risk of Bias 2* (RoB-2) tool for RCTs [21]. This tool comprises five domains that address fundamental aspects of this type of design:

(1) Randomisation process;
(2) Deviations from intended interventions;
(3) Missing outcome data;
(4) Measurement of the outcome;
(5) Selection of the reported result.

Each domain contains multiple signaling questions with the following response options: “Yes,” “Probably yes,” “No,” “Probably no,” and “Not informed.” The answers determine the risk of bias judgment for each domain: “Low,” “Some concerns,” or “High.” The overall risk of bias for each study is rated as “Low” if all domains are scored as such, “Some concerns” if one or more domains receive this rating, or “High” if at least one domain is judged as high-risk.

This assessment was conducted by VSF and PMRM. Inter-rater reliability was high; however, any discrepancies were discussed with CRP until consensus was reached.

### 2.6. Data Extraction

Two reviewers (VSF and PMRM) independently extracted data using a piloted template. The following information was collected for each study: (i) authors, year, and country; (ii) sample size and demographic characteristics; (iii) details of the intervention, including motivational interviewing format, components, and session length; (iv) comparators; (v) follow-up duration; (vi) outcome definitions and statistical results; and (vii) risk of bias domains (RoB-2). Disagreements between reviewers were resolved through discussion and consensus. When necessary, a third reviewer (CRP) adjudicated unresolved discrepancies.

MIs were considered effective among university students when they resulted in reductions in the frequency of alcohol consumption over various time periods, the number of binge drinking episodes, alcohol-related consequences (measured using the *Rutgers Alcohol Problem Index* [RAPI] and the *Young Adult Alcohol Consequences Questionnaire* [YAACQ]), and blood alcohol concentration (g/dL).

### 2.7. Data Analysis

A qualitative synthesis of the included studies was performed, gathering information on the frequency of alcoholic beverage consumption, binge drinking episodes, alcohol-related consequences, and blood alcohol concentration levels reported by university students.

For the quantitative analysis, meta-analyses were conducted for those alcohol-related variables reported in at least three studies. The analysis method used was the inverse variance approach. For each case, means and standard deviations of the outcome variables in the intervention and control groups were used at the end of follow-up after the MI. Statistical heterogeneity among studies was assessed with the I2 statistic. Values of <25%, 26–50%, and >75% were interpreted as low, moderate, and high heterogeneity, respectively. The analyses were performed using *RevMan* version 5.4 (The Cochrane Collaboration, London, UK). All hypothesis tests were two-tailed, with significance set at *p* < 0.05 and a 95% confidence interval.

For each meta-analysis conducted, publication bias risk was assessed by visually inspecting the corresponding funnel plots. Asymmetry was taken to suggest potential publication bias.

## 3. Results

### 3.1. Study Selection

After conducting searches in the PubMed, Scopus, and BVS Library databases, a total of 675 records were identified. Following duplicate removal using Mendeley Reference Manager (Mendeley Ltd., London, UK), 624 records were screened by title and abstract according to the exclusion criteria described in Methods section. As a result, 33 studies were selected for full-text review. Ultimately, 15 studies were included in the qualitative synthesis [17,18,22,23,24,25,26,27,28,29,30,31,32,33,34], and 14 in the quantitative synthesis [17,18,22,23,24,25,27,28,29,30,31,32,33,34]. The selection process is depicted in the PRISMA flow diagram (Figure 1).

### 3.2. Characteristics of the Selected Studies

All included studies applied MIs in comparison with control groups [17,18,22,23,24,25,26,27,28,29,30,31,32,33,34]. The studies comprised a total sample size of 2389 university students, of whom 60.98% were women and 39.02% were men, with ages ranging from 18 to 25 years.

A total of 1529 university students were assigned to the intervention groups, while 860 participants formed part of the various control groups across the different studies. The characteristics of the fifteen included studies are summarized in the results table (Table 2).

### 3.3. Risk of Bias

**Figure 2:**
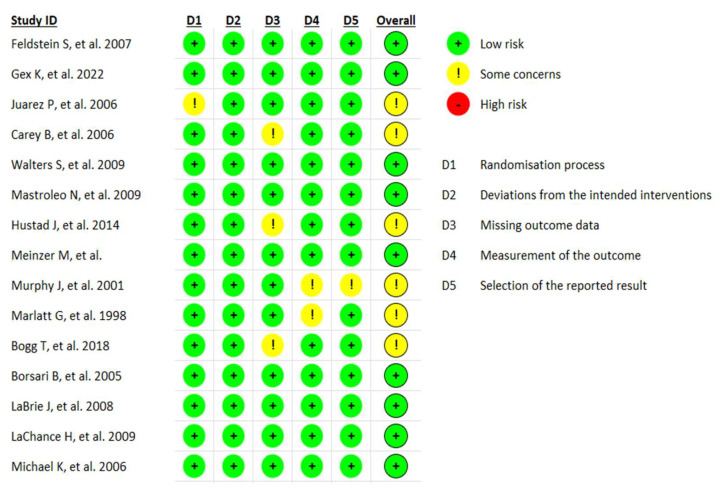
Risk of bias in included RCTs [17,18,22,23,24,25,26,27,28,29,30,31,32,33,34].

The risk of bias was assessed using the RoB-2 tool for RCTs [21]. According to this assessment (Figure 2), nine studies were rated as having a low risk of bias, while six studies were rated as “some concerns”. It is noteworthy that none of the studies included in the present review were judged to have a high risk of bias.

### 3.4. Alcohol Consumption Frequency

Twelve studies evaluated the frequency of alcohol consumption among university students [17,22,23,24,26,27,28,30,31,32,33,34]. DDQ scores decreased significantly in the group receiving MI in two studies [26,33]. However, one study found no statistically significant differences when comparing MI with the control group [34].

**Figure 3:**
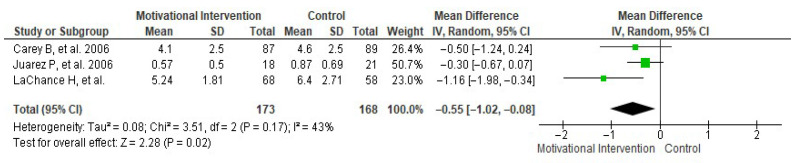
Forest plot showing the effect of MIs on the number of daily drinks consumed [17,23,31].

**Figure 4:**
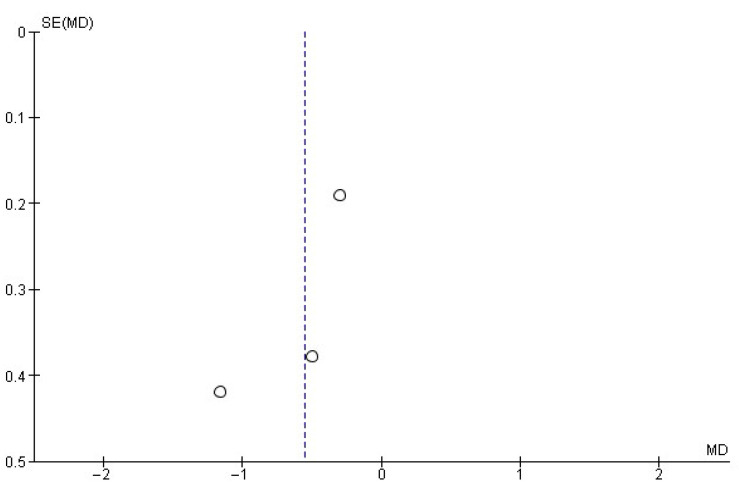
Funnel plot showing the risk of publication bias in studies of MIs on the number of daily drinks consumed.

Regarding the number of daily drinks consumed, a random-effects meta-analysis including three studies [17,23,31] was conducted. In this synthesis (Figure 3), a mean difference of −0.55 ± 0.47 daily drinks was found in favor of MI, with an overall effect test Z = 2.28 (*p* = 0.02) and heterogeneity I^2^ = 43%. The risk of publication bias for this meta-analysis is shown in Figure 4.

**Figure 5:**
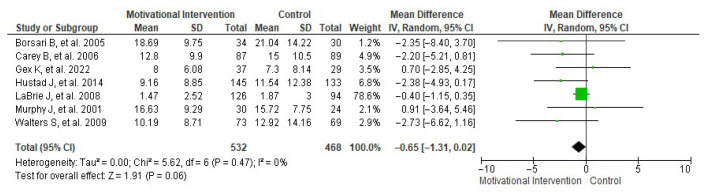
Forest plot showing the effect of MIs on the number of weekly drinks consumed [22,23,24,27,28,30,32].

**Figure 6:**
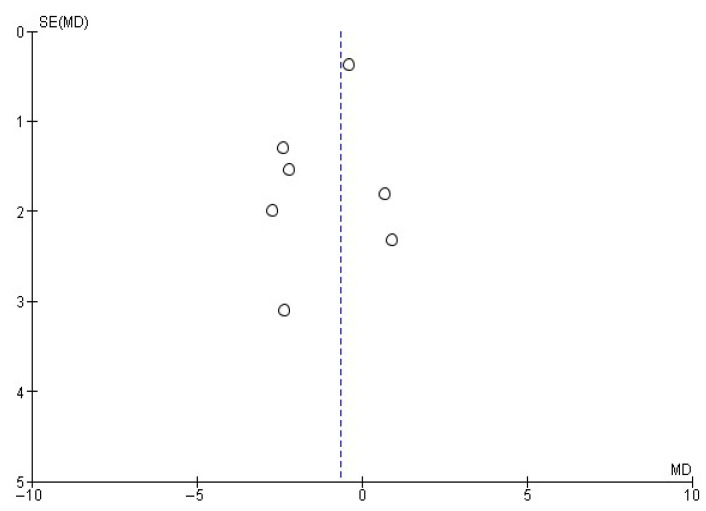
Funnel plot showing the risk of publication bias in studies of MIs on the number of weekly drinks consumed.

Another frequency evaluated by several studies [22,23,24,27,28,30,32] was weekly alcohol consumption. In this case, the random-effects meta-analysis (Figure 5) yielded a mean difference of −0.65 ± 0.66 weekly drinks, with an overall effect test Z = 1.91 (*p* = 0.06) and heterogeneity I^2^ = 0%. Figure 6 shows the publication bias risk related to this meta-analysis.

### 3.5. Alcohol Intoxication Episodes

The number of alcohol intoxication episodes was assessed in seven studies [22,23,28,29,30,32,33]. One study [32] reported significant differences after applying an MI based on the BASICS methodology in weekly intoxication episodes (from 3.29 ± 1.38 to 2.14 ± 0.77), whereas the group receiving assessment only decreased from 3.25 ± 0.97 to 2.49 ± 1.38. However, another study [23] found no significant differences when applying MI (from 7.6 ± 5.2 to 4.9 ± 3.5) compared to the assessment-only group (from 7.7 ± 4.1 to 5.1 ± 4.0). Similarly, another study [29] did not find differences when comparing MI with the control group (from 1.29 ± 1.25 to 0.77 ± 1.06, *p* = 0.45; from 1.67 ± 1.45 to 1.27 ± 0.96, *p* = 0.253).

**Figure 7:**
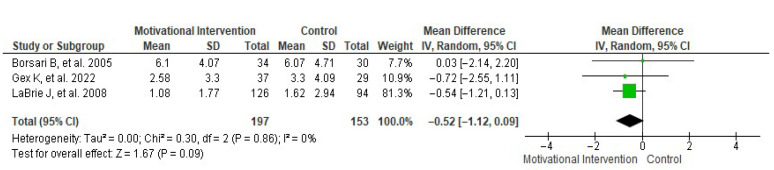
Forest plot showing the effect of MIs on the number of monthly drinks consumed [22,28,30].

**Figure 8:**
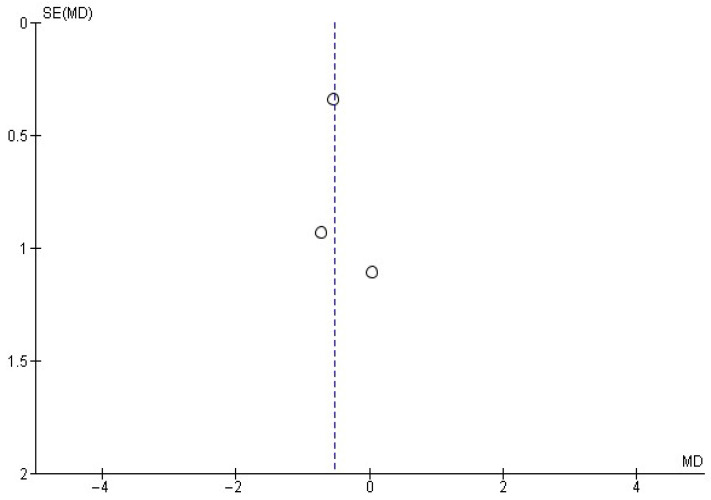
Funnel plot showing the risk of publication bias in studies of MIs on the number of monthly drinks consumed.

Regarding monthly intoxication episodes, data reported by three studies [22,28,30] were included in a random-effects meta-analysis (Figure 7). This analysis showed a mean difference in monthly intoxication episodes of −0.52 ± 0.60, with an overall effect test of *Z* = 1.67 (*p* = 0.09) and heterogeneity of *I*^2^ = 0%. Additionally, Figure 8 shows the publication bias risk for the number of monthly intoxication episodes.

### 3.6. Consequences of Alcohol Consumption

**Figure 9:**
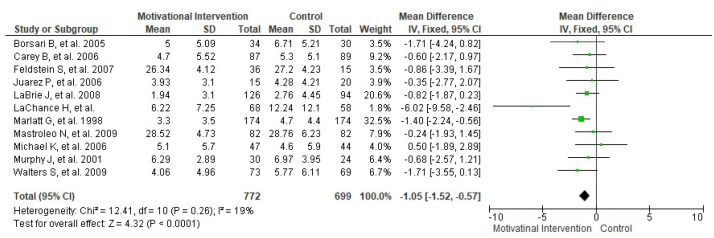
Forest plot showing the effect of MIs on RAPI score [17,18,22,23,27,29,30,31,32,33,34].

**Figure 10:**
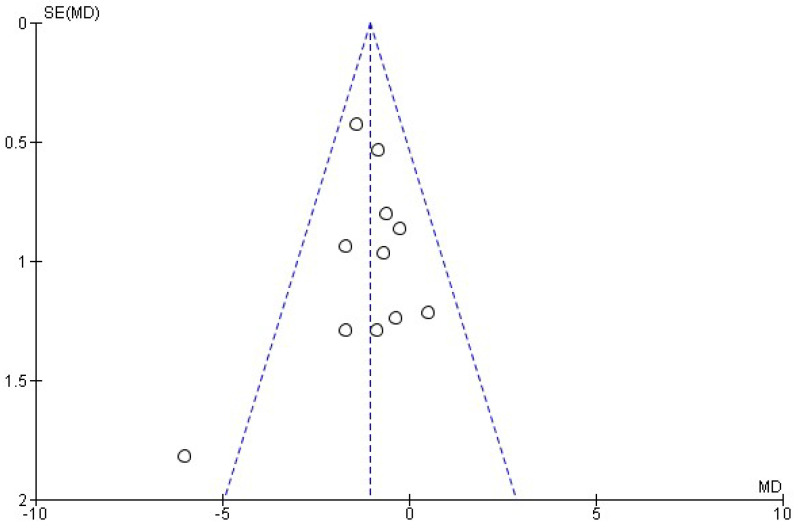
Funnel plot showing the risk of publication in studies of MIs on RAPI score.

The consequences associated with excessive alcohol use were assessed in fourteen studies [17,18,22,23,24,25,27,28,29,30,31,32,33,34]. Specifically, eleven of these [17,18,22,23,27,29,30,31,32,33,34] evaluated the effect of MIs on the RAPI score. Based on the synthesis of this outcome variable, a fixed-effects meta-analysis was performed (Figure 9), showing that MIs produced a mean reduction in RAPI score of −1.05 ± 0.47 points, with an overall effect test of *Z* = 4.32 (*p* < 0.0001) and low heterogeneity (*I*^2^ = 19%). For this meta-analysis, the risk of publication bias was assessed using the corresponding funnel plot (Figure 10).

### 3.7. Blood Alcohol Concentration

**Figure 11:**
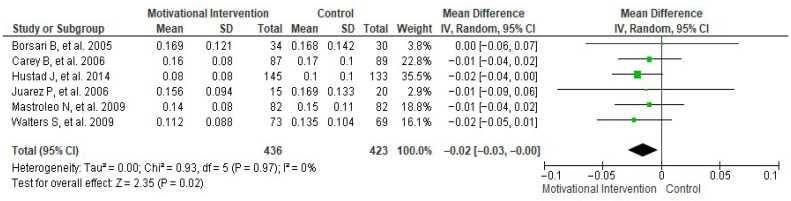
Forest plot showing the effect of MIs on blood alcohol concentration [23,24,27,30,31,33].

**Figure 12:**
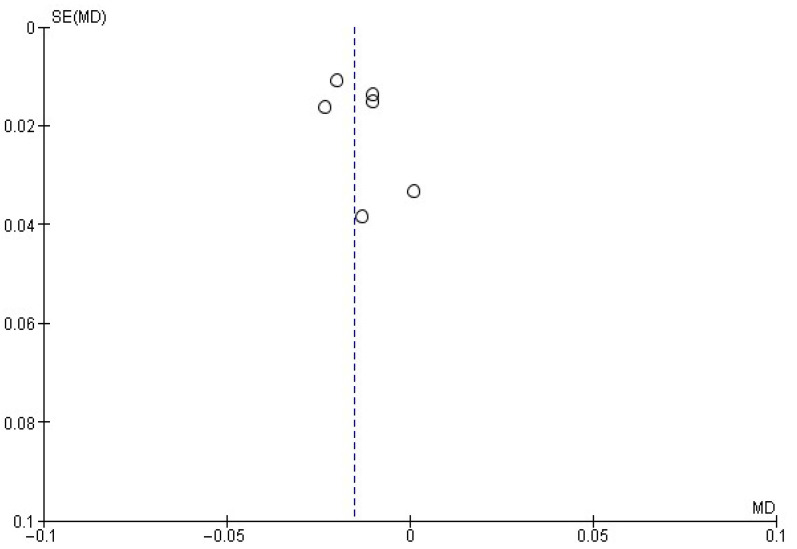
Funnel plot showing the risk of publication bias in studies of MIs on blood alcohol concentration.

Regarding blood alcohol concentration, this variable was reported in six studies [23,24,27,30,31,33]. In this case, the meta-analysis (Figure 11) showed a reduction in blood alcohol concentration of −0.02 ± 0.01 g/dL in favor of MIs. This was associated with an overall effect test of *Z* = 2.35 (*p* = 0.02) and *I*^2^ = 0%. Although statistically significant, the effect size was small, and its clinical relevance remains uncertain. The risk of publication bias is presented in Figure 12.

## 4. Discussion

The meta-analyses showed that MIs achieved small but consistent reductions in daily alcohol consumption, with favorable though non-significant trends at weekly and monthly levels. MIs were also associated with modest reductions in alcohol-related consequences and blood alcohol concentration. These findings suggest that MI can provide incremental benefits in university populations, although the clinical magnitude of these effects remains limited. Some included studies reported significant decreases in quantity and frequency of alcohol use, as well as occasional reductions in episodes of intensive consumption (alcohol intoxication), compared with control groups or standard non-motivational interventions. These results reinforce the importance of behavior change—oriented interventions and the resolution of ambivalence as key strategies for addressing alcohol-related risk behaviors.

The reviewed studies demonstrate that the effectiveness of these interventions can be sustained in the short, medium, and long term. Significant effects were observed at 2–3 months [18,22,26,28,29,31,33], at 6–9 months [17,24,25,27,30,32], and at one year or more after the intervention [23,34]. These findings are consistent with those of previous systematic reviews, which found that MIs generally produced significant effects, although the impact tended to diminish over the long term [35].

It is worth highlighting the brief nature of the interventions, with a mean duration of 68 min per session; the shortest lasted 45 min [29,34], and the longest lasted 3 h [17]. These durations are consistent with a 2015 review [36] of brief interventions aimed at reducing alcohol use in adolescents and young adults, which also concluded that such interventions are effective and beneficial in both the short and long term for reducing alcohol consumption and its adverse effects.

Among the MIs employed, some studies specifically utilized motivational interviewing [25,29,33]. Previous research has demonstrated the effectiveness of MI in reducing substance use among adolescents [37], although some studies, due to risk of bias and low statistical significance, suggest that MI alone may not be sufficiently beneficial in the long term [38]. Other studies incorporated feedback into the intervention [27,31]; feedback reinforces intrinsic motivational processes and acts as an active component that enhances intervention outcomes. Additional evidence from studies not included in this review also concludes that the use of feedback helps reduce both the frequency and quantity of alcohol consumption [39]. Moreover, a meta-analysis conducted within the INTEGRATE project directly evaluated the effect of personalized feedback. Although the results did not show clear increases in motivation to change, they indicated that group modalities combining MI with feedback were the most effective [40]. Finally, another review found that feedback components within brief MIs positively influenced the reduction in alcohol consumption in the medium term [41].

Another strategy that has shown positive results is the combination of MIs with educational components. This integrative approach seeks not only to mobilize internal motivation for change but also to provide essential knowledge about alcohol’s effects, coping strategies, and informed decision-making. Several studies have demonstrated that this combination can be more effective than either approach alone. For example, the systematic review by Foxcroft et al. [42] reported that interventions combining motivational elements with personalized educational content—such as information on risks, standard drink units, and reduction strategies—led to greater reductions in drinking frequency and in negative consequences associated with alcohol use. Similarly, studies by Hustad et al. [24], Gex et al. [28], and Borsari et al. [30], included in this review, showed that when educational content is delivered in an interactive and tailored format, intervention maintains student autonomy while improving knowledge about the risks of excessive alcohol use. This strategy appears particularly useful in university settings, where drinking patterns are often sustained by misconceptions and a low perception of risk.

Regarding the number of drinks consumed per day, the results of the meta-analysis showed a significant reduction of −0.55 drinks per day in the MI group. These findings are consistent with previous meta-analyses [35,37] that have documented similar reductions in average consumption following MI. Furthermore, the fact that the effect size remained significant despite only three studies being included in this synthesis suggests a clear direction of effect. In relation to weekly and monthly alcohol consumption, the meta-analysis showed a mean reduction of −0.65 drinks per week and −0.52 monthly episodes of alcohol intoxication in the groups receiving MI. Although these differences did not reach statistical significance, the results suggest a clinically relevant trend toward reduced consumption. This reduction pattern has also been observed in previous studies [36,37,41,42], which describe that brief MIs tend to generate more pronounced effects on drinking frequency in structured contexts with proper follow-up. It is important to note that reducing just one episode of intoxication per month may have a considerable clinical impact on preventing injuries, risky sexual behavior, and decreased academic performance associated with excessive drinking among university students [43,44].

The pooled analysis of studies evaluating alcohol-related consequences yielded statistically significant results, demonstrating that, beyond reducing frequency and quantity of consumption, MIs also decrease negative outcomes such as interpersonal, academic, legal, and health problems associated with excessive drinking [45,46]. Additionally, the meta-analysis indicated that MIs have a positive effect on blood alcohol concentration among university students. Although the observed reduction may appear modest, even small decreases in blood alcohol levels can meaningfully reduce the risk of accidents, psychomotor impairment, and risk behaviors such as driving under the influence or engaging in unprotected sexual activity [47]. Previous research has shown that students with heavy alcohol use are at increased risk for mental health problems, alcohol use disorders, and physical health issues [48], further underscoring the importance of these findings.

### Limitations and Strengths

This systematic review and meta-analysis have several limitations. First, there was heterogeneity in how MI was implemented across studies. Although all studies employed MI as a central strategy, differences in the interventionist training and in specific components (e.g., use or omission of personalized feedback) may have influenced results and complicated direct comparisons. Likewise, although intervention duration was relatively consistent, variability in follow-up periods introduces bias when assessing the long-term sustainability of effects, as not all studies reported long-term outcomes. Another limitation is that most included studies were conducted in high-income countries, which limits the generalizability of the results to other sociocultural contexts. As with all systematic reviews, the possibility of publication bias cannot be excluded. This was assessed by examining the asymmetry observed in the funnel plots.

Although the included RCTs varied in intervention components, delivery formats, and follow-up durations, comparability was ensured by standardizing outcome metrics across studies and by applying random-effects models in all meta-analyses. This approach minimized the impact of methodological heterogeneity and increased the reliability of the pooled estimates.

Heterogeneity in MI content, interventionist training, session dosage, and follow-up timing likely contributed to between-study variability and should be considered when interpreting the pooled results. Most trials were conducted in high-income Western countries, which limits the external validity and generalizability of our findings to other cultural and socioeconomic contexts. Additionally, reliance on self-reported alcohol consumption introduces measurement bias. The modest number of studies per outcome limited the ability to conduct subgroup and sensitivity analyses and precluded meta-regression, thereby reducing the capacity to identify potential effect modifiers. These factors may limit both the precision and the broader applicability of our conclusions.

Nevertheless, this review has several methodological and analytical strengths that reinforce the validity of the findings. First, the review focused exclusively on MIs applied to university populations, allowing for a precise evaluation of the impact of this strategy in a group particularly vulnerable to excessive alcohol use. Although all interventions had a similar duration, variability in MI delivery provided the opportunity to explore different approaches within the same theoretical framework. Furthermore, the inclusion of studies with short-, medium-, and long-term follow-up enabled assessment of both immediate effects and the potential durability of outcomes over time. The use of a random-effects model in the meta-analysis provided an appropriate statistical approach to address conceptual heterogeneity. Finally, the review process followed rigorous criteria for study search, selection, and critical appraisal, thereby reducing the risk of bias and increasing the reliability of the conclusions.

## 5. Conclusions

MIs produce modest improvements in alcohol-related outcomes among university students. While some effects reached statistical significance, their clinical magnitude was generally small, and interpretation should therefore be made with caution. MI alone is unlikely to generate large changes, but its brevity and feasibility make it a valuable component within broader, multi-component prevention and intervention strategies in university settings.

Future studies should prioritize the use of standardized outcome measures, evaluate longer follow-up periods, and extend research to non-Western and resource-limited contexts to enhance external validity. In addition, cost-effectiveness analyses are needed to inform sustainable implementation and optimize both the equity and clinical relevance of MIs in university populations.

## Figures and Tables

**Table 1:** Comprehensive search strategies.

Database	Search Strategy	Filters Applied
PubMed	(motivational interview OR motivational interventions) AND (alcohol consumption OR alcohol drinking OR alcohol-related disorders) AND (university students OR college students)	Type of study: Randomized Controlled Trials
Scopus	ALL (“motivational interview”) OR ALL (“motivational intervention”) AND TITLE (“alcohol consumption”) OR TITLE (“alcohol drinking”) OR ALL (“alcohol-related disorders”) AND TITLE (“university students”) OR TITLE (“college students”)	Not applicable
BVS Library	(“motivational interview”) OR (“motivational intervention”) AND (“alcohol consumption”) OR (“alcohol drinking”) OR (“alcohol-related disorders”) AND (“university students”) OR (“college students”)	Type of study: Randomized Controlled Trials Language: English and Spanish

**Table 2:** Results table.

**Authors, Year, Country**	**Population**	**Age of Participants**	**Type of Intervention**	**Follow-Up**	**Results**	**Conclusion**	**Risk of Bias**
Feldstein S et al. [29] (2007) USA	51 university students. 36 IG and 15 CG	18–20 years	MI (IG, n = 36) and single assessment (CG, n = 15).	2-month follow-up	Reduction in binge drinking episodes only in IG (t[34] = 2.08, *p* < 0.05). RAPI score decreased in both groups (IG: t[34] = 4.58, *p* < 0.001; CG: t[14] = 4.58, *p* < 0.001)	Motivational interviewing showed greater reductions in binge drinking episodes and alcohol-related problems	Low
Gex K et al. [28] (2022) USA	66 university students. 37 IG and 29 CG	18–25 years	MI + SFAS (IG, n = 37) and alcohol education (CG, n = 29).	3-month follow-up	YAACQ score dropped from 8.30 ± 5.52 to 5.77 ± 5.59 (Cohen’s d = 0.46 [0.136–0.900]) in IG; in CG from 7.50 ± 4.51 to 4.80 ± 3.72 (Cohen’s d = 0.65)	A brief MI combined with SFAS is feasible and well tolerated by university students	Low
Juárez P et al. [31] (2006) USA	89 university students. 68 IG and 21 CG	Mean age 19.43 years	MI (IG, n = 15), MI + in-person feedback (IG, n = 15), MI + online feedback (IG, n = 18), online feedback (IG, n = 20), single assessment (CG, n = 21).	2-month follow-up	Daily drinks: MI from 1.29 ± 1.13 to 0.59 ± 0.52; MI + in-person feedback from 2.12 ± 1.36 to 1.20 ± 1.56; MI + online feedback from 1.42 ± 0.80 to 0.57 ± 0.50; online feedback from 1.77 ± 1.08 to 0.80 ± 0.64; CG from 1.57 ± 1.26 to 0.87 ± 0.69	Reductions in alcohol consumption observed in participants receiving both types of feedback with MI	Some concerns
Carey B et al. [23] (2006) USA	262 university students. 173 IG and 89 CG	18–25 years	MI (IG, n = 87); enhanced MI with decisional balance module (IG, n = 86); single assessment (CG, n = 89).	12-month follow-up	Daily drinks: MI from 5.7 ± 3.4 to 4.1 ± 2.5; enhanced MI from 5.8 ± 3.3 to 4.5 ± 2.2; CG from 5.8 ± 2.6 to 4.6 ± 2.5. Weekly binge episodes: MI from 7.6 ± 5.2 to 4.9 ± 3.5; enhanced MI from 7.0 ± 4.2 to 5.7 ± 4.2; CG from 7.7 ± 4.1 to 5.1 ± 4.0	Alcohol consumption decreases with MI tailored for heavy-drinking university students	Some concerns
Walters S et al. [27] (2009) USA	279 university students. 210 IG and 69 CG	Mean age 19.8 years	MI (IG, n = 70); MI + feedback (IG, n = 73); feedback (IG, n = 67); single assessment (CG, n = 69).	6-month follow-up	Significant changes in weekly consumption at follow-up (t[275] = −2.11, *p* = 0.04). BAC levels decreased by 79% in IG	MI combined with feedback is effective in reducing alcohol consumption and related problems	Low
Mastroleo N et al. [33] (2010) USA	238 university students. 156 IG and 82 CG	18–20 years	BASICS-based motivational sessions. EAA (IG, n = 74); CPA (IG, n = 82); single assessment (CG, n = 82).	3-month follow-up	Binge episode scores decreased significantly in BASICS groups: EAA from −0.58 ± 2.70 to −1.07 ± 3.08; CPA from 0.52 ± 2.99 to −0.58 ± 2.66; CG from −0.30 ± 3.34 to 0.05 ± 3.59	BASICS-based MI was effective in reducing excessive alcohol consumption	Low
Hustad J et al. [24] (2014) USA	178 university students. 145 IG and 133 CG	Mean age 19.08 years	Group MI (IG, n = 145); individual MI (CG, n = 133).	6-month follow-up	YAACQ: group MI from 5.59 ± 4.94 to 3.30 ± 6.01; individual MI from 6.19 ± 5.41 to 3.84 ± 6.41. Weekly drinks: group MI from 5.59 ± 4.94 to 3.30 ± 6.01; individual MI from 6.19 ± 5.41 to 3.84 ± 6.41	Both individual and group MI reduced alcohol consumption in university students	Some concerns
Meinzer M et al. [26] (2021) USA	113 university students. 55 IG and 58 CG	Mean age 19.87 years	MI + behavioral activation (IG, n = 55); MI + supportive counseling (CG, n = 58).	3-month follow-up	DDQ: IG from 16.10 to 10.94; CG from 13.75 to 8.63. YAACQ: IG from 7.02 to 5.92; CG from 5.42 to 4.78	Adding behavioral activation to MI reduces alcohol-related consequences	Low
Murphy J et al. [32] (2001) USA	39 university students. 27 IG and 12 CG	Mean age 19.6 years	BASICS (IG, n = 14); alcohol education (IG, n = 14); single assessment (CG, n = 12).	9-month follow-up	Weekly drinking days: BASICS from 31.79 ± 11.57 to 21.36 ± 10.08; education from 27.75 ± 9.32 to 25.98 ± 14.38; CG from 29.25 ± 7.73 to 19.85 ± 6.69	BASICS-based MI benefits excessive drinking reduction in university students	Some concerns
Marlatt G et al. [34] (1998) USA	348 university students. 174 IG and 174 CG	18–19 years	MI (IG, n = 174); single assessment (CG, n = 174).	2-year follow-up	DDQ: IG from 4.7 ± 2.3 to 3.6 ± 2.5; CG from 4.2 ± 2.7 to 4.0 ± 2.8. ADS: IG from 7.9 ± 3.8 to 6.5 ± 3.5; CG from 8.2 ± 3.9 to 7.8 ± 4.5	MI significantly reduces alcohol consumption in university students	Some concerns
Bogg T et al. [25] (2018) USA	145 university students. 93 IG and 52 CG	Mean age 20.4 years	BASICS (IG, n = 44); BASICS + educational commitment (IG, n = 49); alcohol education (CG, n = 52).	9-month follow-up	BDP drinking frequency: BASICS from 2.93 ± 1.21 to 1.87 ± 1.39; BASICS+educational commitment from 2.80 ± 1.08 to 2.08 ± 1.38; CG from 3.02 ± 1.42 to 2.50 ± 1.67	BASICS-based MI is effective as a strategy to address alcohol consumption	Some concerns
Borsari B et al. [30] (2005) USA	64 university students. 34 IG and 30 CG	Mean age 19.1 years	MI (IG, n = 34); alcohol education (CG, n = 30).	6-month follow-up	Weekly drinks: MI from 19.22 ± 9.65 to 18.69 ± 9.75; CG from 20.95 ± 10.33 to 21.04 ± 14.22. RAPI: MI from 9.88 ± 7.81 to 5.00 ± 5.09; CG from 7.00 ± 4.84 to 6.71 ± 5.21	Both groups reduced alcohol use, but MI showed greater benefits in reducing alcohol-related problems	Low
LaBrie J et al. [22] (2008) USA	220 female university students. 126 IG and 94 CG	Mean age 18.1 years	Group MI (IG, n = 126); single assessment (CG, n = 94).	10-week follow-up	Fewer binge drinking episodes in IG at follow-up (F[1, 202] = 8.75, *p* < 0.01). Alcohol-related consequences were also lower in IG (F[1, 193] = 3.90, *p* = 0.05)	MI participants showed lower alcohol use and consequences	Low
LaChance H et al. [17] (2009) USA	206 university students. 148 IG and 58 CG	Mean age 18.6 years	Enhanced group MI (IG, n = 68); FAC education (IG, n = 80); alcohol education (CG, n = 58).	6-month follow-up	Daily drinks: group MI from 5.98 ± 2.40 to 5.24 ± 1.81; FAC from 6.00 ± 2.25 to 5.95 ± 2.15; CG from 6.15 ± 2.37 to 6.40 ± 2.71	MI showed greater benefits compared to other groups in reducing alcohol consumption	Low
Michael K et al. [18] (2006) USA	91 university students. 47 IG and 44 CG	Mean age 18.5 years	MI (IG, n = 47); single assessment (CG, n = 44).	45-day follow-up	Weekly drinking days in MI group remained at 5.3; CG from 5.9 to 5.8. Binge episodes: MI from 4.1 ± 5.2 to 2.7 ± 3.2; CG from 4.4 ± 5.1 to 4.2 ± 5.3	MI could feasibly be incorporated into strategies to address alcohol consumption in university students	Low

Abbreviations: IG: Intervention Group; CG: Control Group; MI: Motivational Intervention; RAPI: Rutgers Alcohol Problem Index (0–69 points, higher scores indicate more problematic drinking); SFAS: Substance-Free Activity Session; YAACQ: Young Adult Alcohol Consequences Questionnaire (0–48 points, higher scores indicate more alcohol-related consequences); BASICS: Brief Alcohol Screening and Intervention for College Students; EAA: Evidence-based Application Approach; CPA: Common Practice Approach; DDQ: Daily Drinking Questionnaire (no fixed score range, higher scores indicate higher weekly drinking frequency); ADS: Alcohol Dependence Scale (0–47 points, higher scores indicate greater alcohol dependence); BDP: Brief Drinker Profile; FAC: Focus on Alcohol Concerns.
